# Supplementary material for: A spectrum of routing strategies for brain networks
Source: PLoS Comput Biol. 2019 Mar 8;15(3):e1006833. doi: 10.1371/journal.pcbi.1006833 (PMC6426276; doi:10.1371/journal.pcbi.1006833)
Supplement: S1 Fig — (PDF) [file pcbi.1006833.s001.pdf]

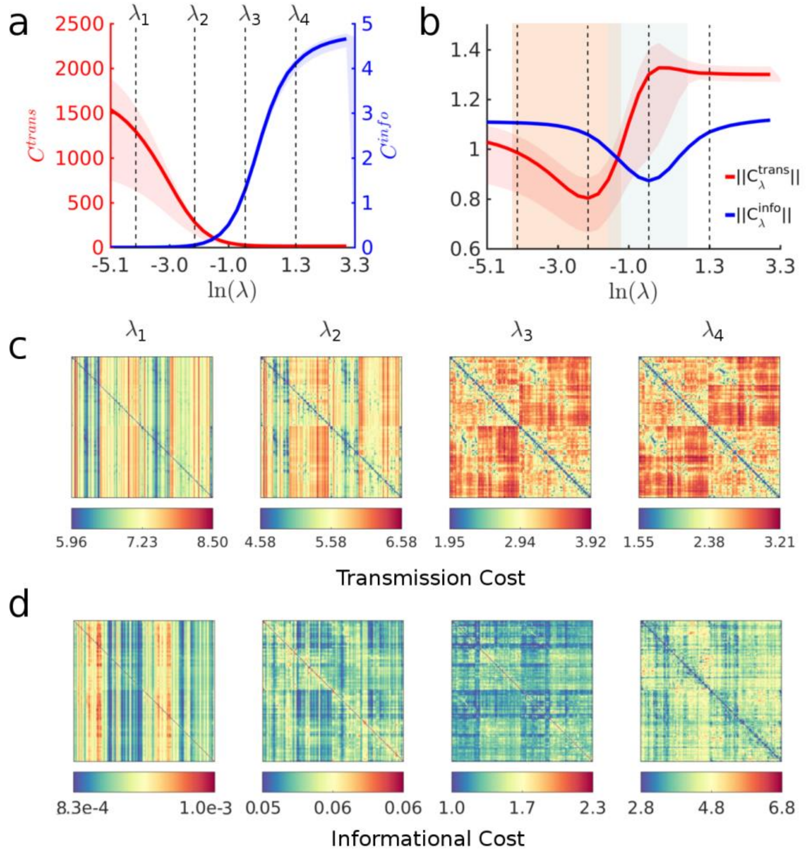

S1 Fig. A spectrum of communication processes. (a) Averages of C_λ^{trans} (red) and C_λ^{info}(blue) across all node pairs, as a function of λ. Solid red and blue lines correspond to the median across all subjects, whereas the shaded red and blue regions denote the 95th percentile. Vertical dashed lines correspond to the values λ_1=$e^{-4.19}$, λ_2=$e^{-2.16}$, λ_3=e-^{042} and λ_4=,$e^{1.31}$. (b) Averages of $\left\|C_\lambda^{trans}\right\|$ (red) and $\left\|C_\lambda^{info}\right\|$ (blue) across all node pairs. These curves are computed by normalizing C_λ^{trans} and C_λ^{info} with respect to the same cost measures computed on ensembles of 500 randomized networks (per subject). Shaded red and blue areas indicate sections of the curves $\left\|C_\lambda^{trans}\right\|$ and $\left\|C_\lambda^{info}\right\|$ that are smaller than 1, respectively, indicating the regions in the spectrum where the communication cost of empirical networks is smaller than the cost computed on the randomized ensembles. The dashed vertical lines are placed at the minimum and maximum of $\left\|C_\lambda^{trans}\right\|$ (λ_2 and λ_3, respectively), and at two points near the extremes of the spectrum ((λ_1 and λ_4). (c) pairwise values of $C_\lambda^{trans}(i,t)$ for all node pairs. (d) pairwise values of $C_\lambda^{info}(i,t)$ for all node pairs. For the panels λ_1=$e^{-4.19}$, λ_2=$e^{-2.16}$, λ_3=e-^{042} and λ_4=$e^{1.31}$.
