# Supplementary material for: A spectrum of routing strategies for brain networks
Source: PLoS Comput Biol. 2019 Mar 8;15(3):e1006833. doi: 10.1371/journal.pcbi.1006833 (PMC6426276; doi:10.1371/journal.pcbi.1006833)
Supplement: S2 Fig — (PDF) [file pcbi.1006833.s002.pdf]

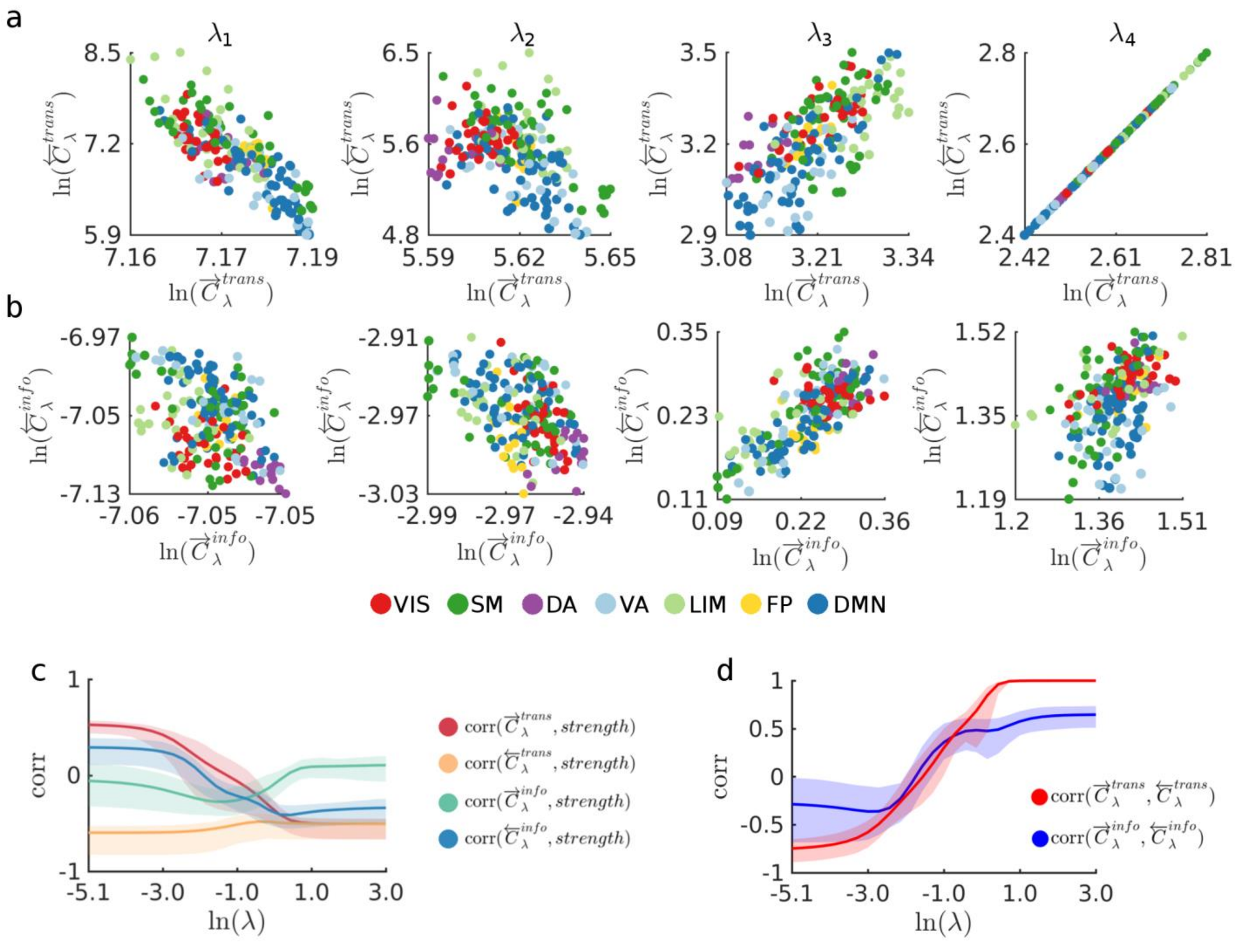

S2 Fig. Nodal average transmission costs for four increasingly biased routing strategies. (a) Scatter plots show the transmission cost associated to each node when it acts as source ($\vec{C}_{\lambda}^{trans}$) and target ($\overleftarrow{C}_{\lambda}^{trans}$) during communication processes taking place under routing strategies generated with the values $\lambda_1=e^{-4.19}$, $\lambda_2=e^{-2.16}$, $\lambda_3=e\text{-}^{042}$ and $\lambda_4=e^{1.31}$. (b) Scatter plots show the transmission cost associated to each node when it acts as source ($\vec{C}_{\lambda}^{info}$) and target ($\overleftarrow{C}_{\lambda}^{info}$) during communication processes taking place under routing strategies generated with the values λ_1, λ_2, λ_3 and λ_4. Markers in the scatter plots in (a) and (b), representing each node, are colored according to the node's functional role according to the 7 intrinsic connectivity networks (ICN) defined by Yeo et al. (2011) [71]: Visual (VIS), Somatomotor (SM), Dorsal Attention (DA), Ventral Attention (VA), Limbic (LIM), Frontal Parietal (FP), and Default Mode Network (DMN) (see Figure SI1 showing ICNs projected on a cortical surface). The size of the markers is proportional to node's strength. (c) Correlations between node strength and $\vec{C}_{\lambda}^{trans}$ (red), $\overleftarrow{C}_{\lambda}^{trans}$(orange), $\vec{C}_{\lambda}^{info}$ (green) and $\overleftarrow{C}_{\lambda}^{info}$ (blue) as a function of λ. Solid lines show median correlation across all subjects, shaded areas surrounding the lines show 95th percentile. Shaded colored areas between the vertical dashed lines indicate regions where the correlations were not significant ($p > 0.001$). (d) Correlation between $\vec{C}_{\lambda}^{trans}$ and $\overleftarrow{C}_{\lambda}^{trans}$(red), and $\vec{C}_{\lambda}^{info}$ and $\overleftarrow{C}_{\lambda}^{info}$ (blue), as a function of λ. Solid lines show medians across all subjects and shaded areas surrounding solid lines show the 95th percentile. Shaded areas between the vertical dashed lines indicate areas where correlation values were not significant ($p > 0.001$).
