# Supplementary material for: A spectrum of routing strategies for brain networks
Source: PLoS Comput Biol. 2019 Mar 8;15(3):e1006833. doi: 10.1371/journal.pcbi.1006833 (PMC6426276; doi:10.1371/journal.pcbi.1006833)
Supplement: S3 Fig — (PDF) [file pcbi.1006833.s003.pdf]

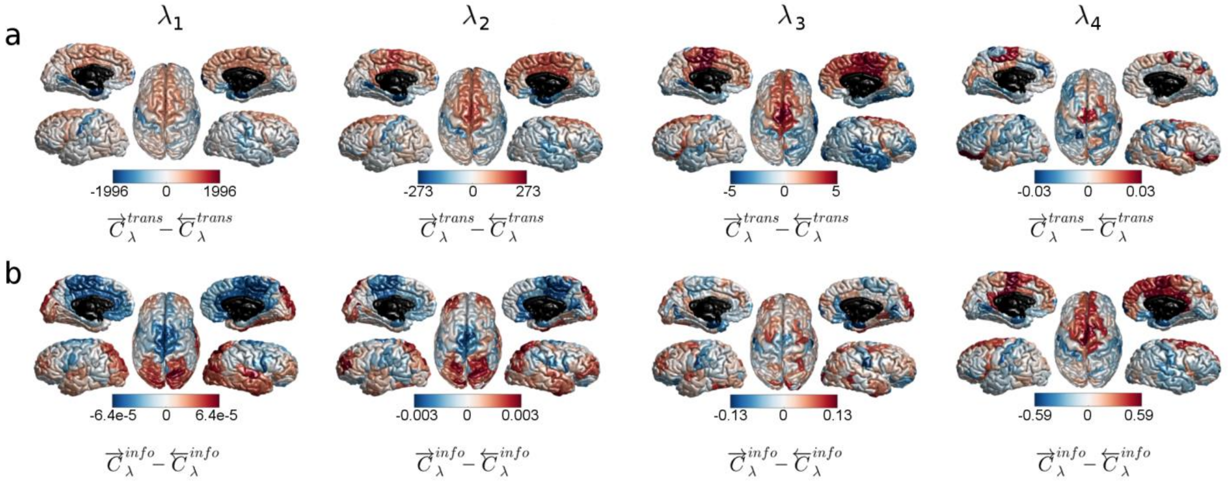

S3 Fig. A brain region's propensity to be a costly source or target. Cortical surfaces show the difference between a node's source and target transmission costs. (a) $\vec{C}_\lambda^{trans} - \overleftarrow{C}_\lambda^{trans}$ for routing strategies generated with the values λ_1, λ_2, λ_3 and λ_4. (b) $\vec{C}_\lambda^{info} - \overleftarrow{C}_\lambda^{info}$ for routing strategies generated with the values λ_1, λ_2, λ_3 and λ_4. Red colored areas on the cortical surfaces correspond to nodes whose source transmission/informational cost is higher than their target transmission/informational cost. Blue colored areas correspond to nodes whose target transmission/informational cost is higher than their source transmission/informational cost. For all panels, $\lambda_1=e^{-4.19}$, $\lambda_2=e^{-2.16}$, $\lambda_3=e\text{-}^{042}$ and $\lambda_4=e^{1.31}$.
