# Supplementary material for: A spectrum of routing strategies for brain networks
Source: PLoS Comput Biol. 2019 Mar 8;15(3):e1006833. doi: 10.1371/journal.pcbi.1006833 (PMC6426276; doi:10.1371/journal.pcbi.1006833)
Supplement: S4 Fig — (PDF) [file pcbi.1006833.s004.pdf]

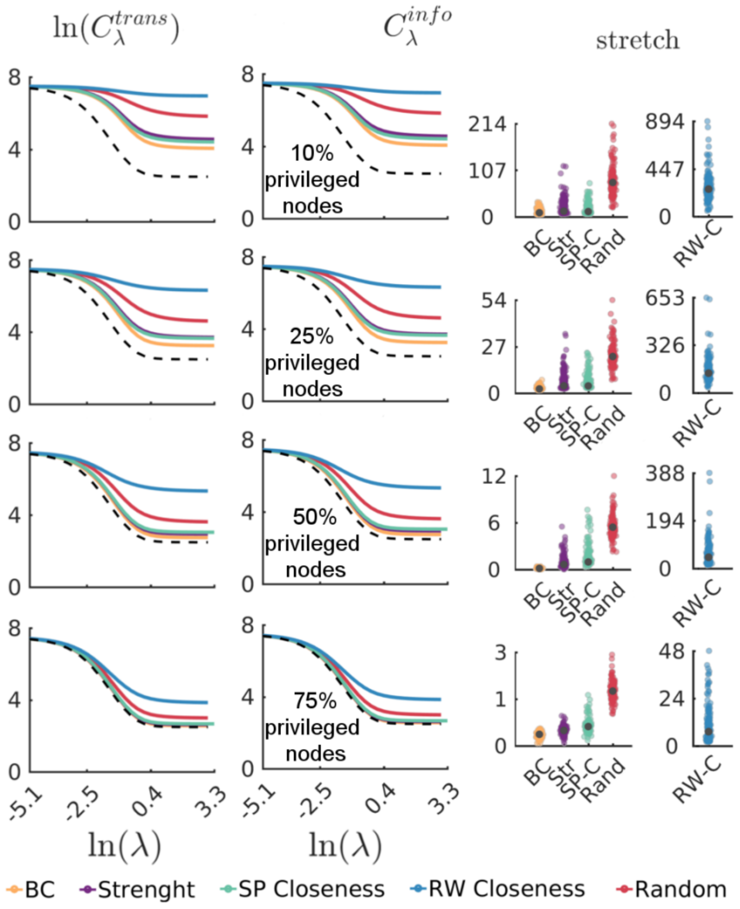


**S4 Fig. Network average values of $C_\lambda^{trans}$ (left panel) and $C_\lambda^{info}$ (middle panel) as a function of λ.** Node medians across all subjects are shown for 22, 55, 110, and 165 *privileged nodes* (corresponding to 10%, 25%, 50% and 75% or the network's nodes) that are selected according to betweenness centrality ranking (yellow line), strength ranking (purple line), shortest-path-based closeness centrality (green line), and random-walk-based closeness centrality (blue line). For comparison purposes, we also show cost measures for randomly sampled nodes (red line). The dotted lines show $C_\lambda^{trans}$ and $C_\lambda^{info}$, respectively, for the case in which all nodes' routing strategies are biased (i.e. 100% privileged nodes). Right panel shows node stretch distributions for the different sets of privileged nodes and centrality rankings. Black markers indicate the median of the distributions.
