# Supplementary material for: A spectrum of routing strategies for brain networks
Source: PLoS Comput Biol. 2019 Mar 8;15(3):e1006833. doi: 10.1371/journal.pcbi.1006833 (PMC6426276; doi:10.1371/journal.pcbi.1006833)
Supplement: S5 Fig — (PDF) [file pcbi.1006833.s005.pdf]

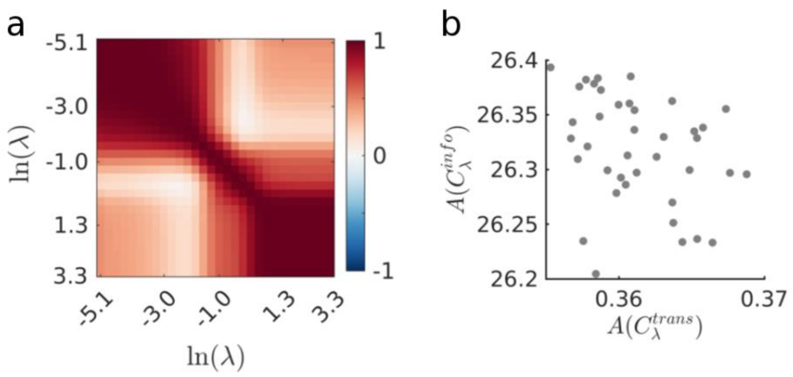

S5 Fig. Communication cost trade-off within subjects. (a) Correlations between all subject's C_λ^{trans} across all values of λ. Positive correlations are colored in red, negative correlations are colored in blue. (b) Scatter plot of the computed areas under the normalized C_λ^{trans} and C_λ^{info} curves, showing a trade-off between the decay of C_λ^{trans} and the growth of C_λ^{info} (correlation between A(C_λ^{trans}) and A(C_λ^{info}) is $r = -0.6$, $p < 0.001$).
