# Supplementary material for: A spectrum of routing strategies for brain networks
Source: PLoS Comput Biol. 2019 Mar 8;15(3):e1006833. doi: 10.1371/journal.pcbi.1006833 (PMC6426276; doi:10.1371/journal.pcbi.1006833)
Supplement: S7 Fig — (PDF) [file pcbi.1006833.s007.pdf]

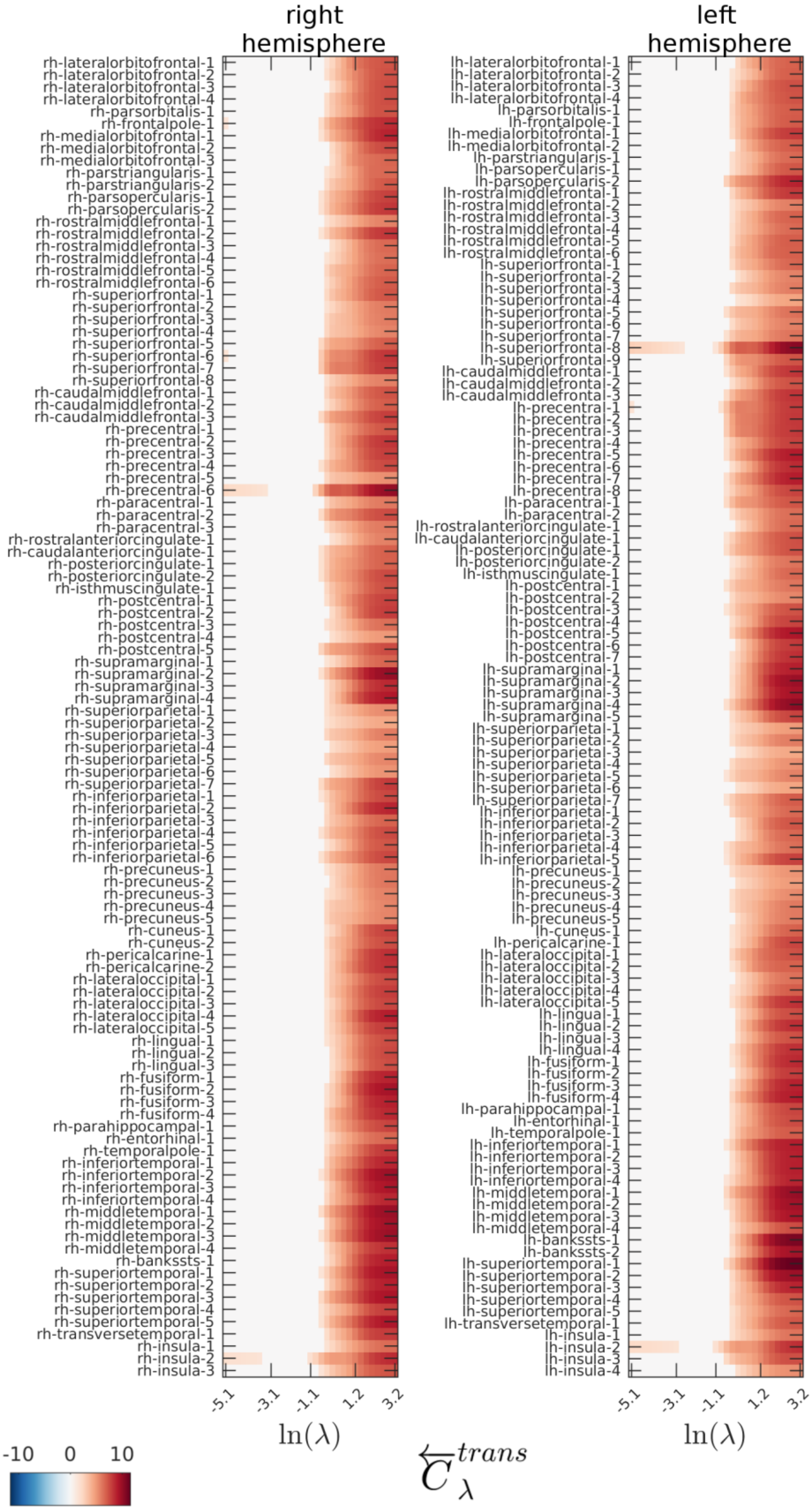
right hemisphere
left hemisphere
-5.1
-3.1
-1.1
1.2
3.2
ln(λ)
-10
0
10
$\overleftarrow{C}_{\lambda}^{trans}$

S7 Fig. z-scored target transmission costs as a function of λ. For each subject's structural connectivity network, the target transmission cost ($\overleftarrow{C}_{\lambda}^{trans}$) of every node was standarized with respect to the corresponding distribution of $\overleftarrow{C}_{\lambda}^{trans}$ measured on an ensemble of 500 randomized networks. z-scored values were then thresholded according to a type I error $\alpha = 0.01$. Red regions on the spectrum indicate nodes with significantly high $\overleftarrow{C}_{\lambda}^{trans}$, whereas blue regions on the spectrum indicate nodes with significantly low $\overleftarrow{C}_{\lambda}^{trans}$.
