# Supplementary material for: A spectrum of routing strategies for brain networks
Source: PLoS Comput Biol. 2019 Mar 8;15(3):e1006833. doi: 10.1371/journal.pcbi.1006833 (PMC6426276; doi:10.1371/journal.pcbi.1006833)
Supplement: S9 Fig — (PDF) [file pcbi.1006833.s009.pdf]

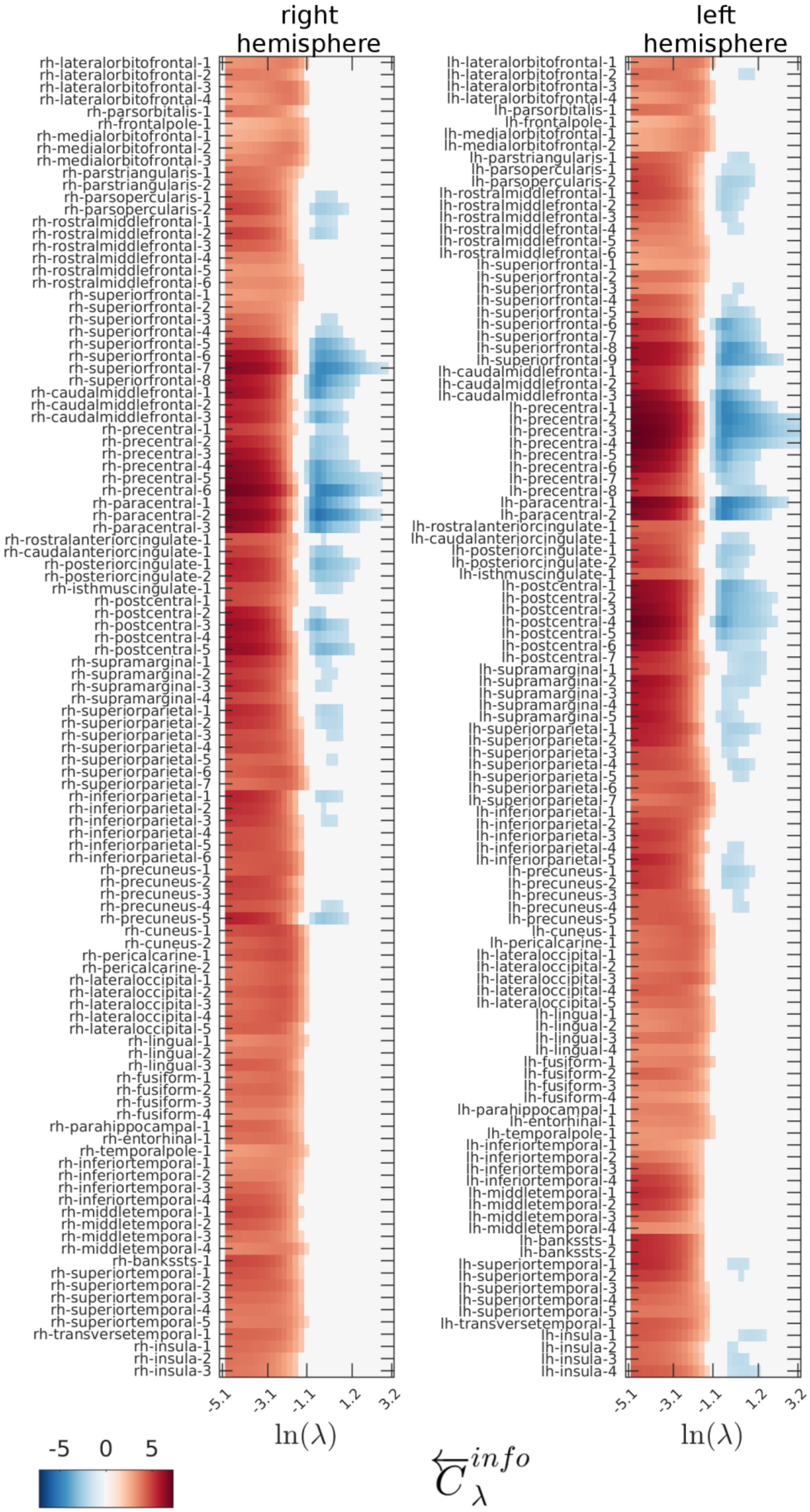
right
hemisphere
rh-lateralorbitofrontal-1
rh-lateralorbitofrontal-2
rh-lateralorbitofrontal-3
rh-lateralorbitofrontal-4
rh-parsorbitalis-1
rh-frontalpole-1
rh-medialorbitofrontal-1
rh-medialorbitofrontal-2
rh-medialorbitofrontal-3
rh-parstriangularis-1
rh-parstriangularis-2
rh-parsopercularis-1
rh-parsopercularis-2
rh-rostralmiddlefrontal-1
rh-rostralmiddlefrontal-2
rh-rostralmiddlefrontal-3
rh-rostralmiddlefrontal-4
rh-rostralmiddlefrontal-5
rh-rostralmiddlefrontal-6
rh-superiorfrontal-1
rh-superiorfrontal-2
rh-superiorfrontal-3
rh-superiorfrontal-4
rh-superiorfrontal-5
rh-superiorfrontal-6
rh-superiorfrontal-7
rh-superiorfrontal-8
rh-caudalmiddlefrontal-1
rh-caudalmiddlefrontal-2
rh-caudalmiddlefrontal-3
rh-precentral-1
rh-precentral-2
rh-precentral-3
rh-precentral-4
rh-precentral-5
rh-precentral-6
rh-paracentral-1
rh-paracentral-2
rh-paracentral-3
rh-rostralanteriorcingulate-1
rh-caudalanteriorcingulate-1
rh-posteriorcingulate-1
rh-posteriorcingulate-2
rh-isthmuscingulate-1
rh-postcentral-1
rh-postcentral-2
rh-postcentral-3
rh-postcentral-4
rh-postcentral-5
rh-supramarginal-1
rh-supramarginal-2
rh-supramarginal-3
rh-supramarginal-4
rh-superiorparietal-1
rh-superiorparietal-2
rh-superiorparietal-3
rh-superiorparietal-4
rh-superiorparietal-5
rh-superiorparietal-6
rh-superiorparietal-7
rh-inferiorparietal-1
rh-inferiorparietal-2
rh-inferiorparietal-3
rh-inferiorparietal-4
rh-inferiorparietal-5
rh-inferiorparietal-6
rh-precuneus-1
rh-precuneus-2
rh-precuneus-3
rh-precuneus-4
rh-precuneus-5
rh-cuneus-1
rh-cuneus-2
rh-pericalcarine-1
rh-pericalcarine-2
rh-lateraloccipital-1
rh-lateraloccipital-2
rh-lateraloccipital-3
rh-lateraloccipital-4
rh-lateraloccipital-5
rh-lingual-1
rh-lingual-2
rh-lingual-3
rh-fusiform-1
rh-fusiform-2
rh-fusiform-3
rh-fusiform-4
rh-parahippocampal-1
rh-entorhinal-1
rh-temporalpole-1
rh-inferiortemporal-1
rh-inferiortemporal-2
rh-inferiortemporal-3
rh-inferiortemporal-4
rh-middletemporal-1
rh-middletemporal-2
rh-middletemporal-3
rh-middletemporal-4
rh-bankssts-1
rh-superiortemporal-1
rh-superiortemporal-2
rh-superiortemporal-3
rh-superiortemporal-4
rh-superiortemporal-5
rh-transversetemporal-1
rh-insula-1
rh-insula-2
rh-insula-3
-5.1
-3.1
-1.1
1.2
3.2
ln(λ)
left
hemisphere
lh-lateralorbitofrontal-1
lh-lateralorbitofrontal-2
lh-lateralorbitofrontal-3
lh-lateralorbitofrontal-4
lh-parsorbitalis-1
lh-frontalpole-1
lh-medialorbitofrontal-1
lh-medialorbitofrontal-2
lh-parstriangularis-1
lh-parsopercularis-1
lh-parsopercularis-2
lh-rostralmiddlefrontal-1
lh-rostralmiddlefrontal-2
lh-rostralmiddlefrontal-3
lh-rostralmiddlefrontal-4
lh-rostralmiddlefrontal-5
lh-rostralmiddlefrontal-6
lh-superiorfrontal-1
lh-superiorfrontal-2
lh-superiorfrontal-3
lh-superiorfrontal-4
lh-superiorfrontal-5
lh-superiorfrontal-6
lh-superiorfrontal-7
lh-superiorfrontal-8
lh-superiorfrontal-9
lh-caudalmiddlefrontal-1
lh-caudalmiddlefrontal-2
lh-caudalmiddlefrontal-3
lh-precentral-1
lh-precentral-2
lh-precentral-3
lh-precentral-4
lh-precentral-5
lh-precentral-6
lh-precentral-7
lh-precentral-8
lh-paracentral-1
lh-paracentral-2
lh-rostralanteriorcingulate-1
lh-caudalanteriorcingulate-1
lh-posteriorcingulate-1
lh-posteriorcingulate-2
lh-isthmuscingulate-1
lh-postcentral-1
lh-postcentral-2
lh-postcentral-3
lh-postcentral-4
lh-postcentral-5
lh-postcentral-6
lh-postcentral-7
lh-supramarginal-1
lh-supramarginal-2
lh-supramarginal-3
lh-supramarginal-4
lh-supramarginal-5
lh-superiorparietal-1
lh-superiorparietal-2
lh-superiorparietal-3
lh-superiorparietal-4
lh-superiorparietal-5
lh-superiorparietal-6
lh-superiorparietal-7
lh-inferiorparietal-1
lh-inferiorparietal-2
lh-inferiorparietal-3
lh-inferiorparietal-4
lh-inferiorparietal-5
lh-precuneus-1
lh-precuneus-2
lh-precuneus-3
lh-precuneus-4
lh-precuneus-5
lh-cuneus-1
lh-pericalcarine-1
lh-lateraloccipital-1
lh-lateraloccipital-2
lh-lateraloccipital-3
lh-lateraloccipital-4
lh-lateraloccipital-5
lh-lingual-1
lh-lingual-2
lh-lingual-3
lh-lingual-4
lh-fusiform-1
lh-fusiform-2
lh-fusiform-3
lh-fusiform-4
lh-parahippocampal-1
lh-entorhinal-1
lh-temporalpole-1
lh-inferiortemporal-1
lh-inferiortemporal-2
lh-inferiortemporal-3
lh-inferiortemporal-4
lh-middletemporal-1
lh-middletemporal-2
lh-middletemporal-3
lh-middletemporal-4
lh-bankssts-1
lh-bankssts-2
lh-superiortemporal-1
lh-superiortemporal-2
lh-superiortemporal-3
lh-superiortemporal-4
lh-superiortemporal-5
lh-transversetemporal-1
lh-insula-1
lh-insula-2
lh-insula-3
lh-insula-4
-5.1
-3.1
-1.1
1.2
3.2
ln(λ)
-5
0
5
$\overleftarrow{C}_{\lambda}^{info}$

**S9 Fig. z-scored target informational costs as a function of λ.** For each subject's structural connec9ivity network, the target informational cost ($\overleftarrow{C}_{\lambda}^{info}$) of every node was standarized with respect to the corresponding distribution of $\overleftarrow{C}_{\lambda}^{info}$ measured on an ensemble of 500 randomized networks. z-scored values were then thresholded according to a type I error $\alpha = 0.01$. Red regions on the spectrum indicate nodes with significantly high $\overleftarrow{C}_{\lambda}^{info}$, whereas blue regions on the spectrum indicate nodes with significantly low $\overleftarrow{C}_{\lambda}^{info}$.
