# Supplementary material for: A spectrum of routing strategies for brain networks
Source: PLoS Comput Biol. 2019 Mar 8;15(3):e1006833. doi: 10.1371/journal.pcbi.1006833 (PMC6426276; doi:10.1371/journal.pcbi.1006833)
Supplement: S1 Text — (PDF) [file pcbi.1006833.s010.pdf]

S10 Supporting Information. Comparison between the nodal C_λ^{trans} measures ($\vec{C}_\lambda^{trans}$ and $\overleftarrow{C}_\lambda^{trans}$) and communicability, betweenness centrality, page rank centrality and clustering coefficient.

The following figures show the medians and 95 percent confidence interval across all subjects of the HCP (FIGS13) and LAU (FIGS14) datasets of the correlation between communicability (comm), betweenness centrality (BC), page rank (PR) and clustering coefficient (CC), and $\vec{C}_\lambda^{trans}$ (panel a) and $\overleftarrow{C}_\lambda^{trans}$ (panel b), as a function of λ. Noticing that some of these curves are similar to the ones showed in Figure 3 (panel c) in the main manuscript, we've also computed the correlation between communicability (comm), betweenness centrality (BC), page rank (PR) and clustering coefficient (CC), and nodal strength (shown here in panel c). This analysis shows that communicability, betweenness centrality and page rank centrality are all highly correlated to the nodal strength, and therefore, they are also highly correlated and anticorrelated to C_λ^{trans} at the extremes of the spectrum. Conversely, the clustering coefficient, which is not correlated to nodal strength, exhibits a different relationship with C_λ^{trans}. These results support the idea of exploring the communication regimes corresponding to the middle regions of the spectrum, as they depart from the typically degree-driven or degree-correlated measures of communication and centrality.

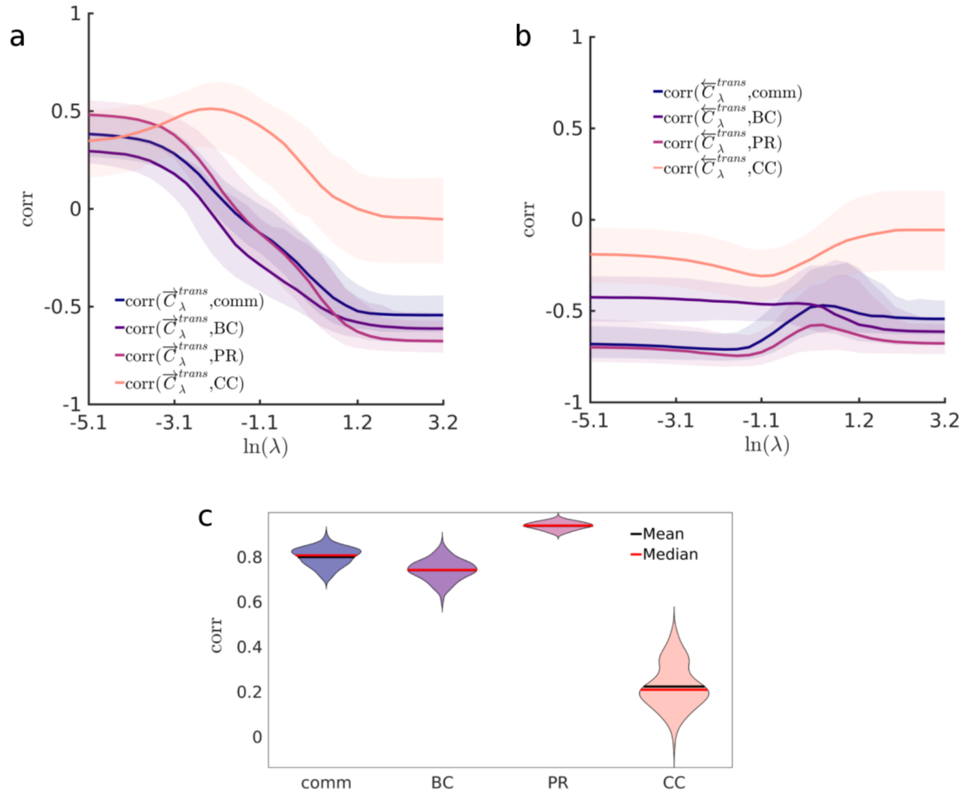

Comparison between the nodal C_λ^{trans} measures ($\vec{C}_\lambda^{trans}$ and $\overleftarrow{C}_\lambda^{trans}$) computed on the HCP dataset, and communicability (comm), betweenness centrality (BC), page rank centrality (PR) and clustering coefficient (CC).

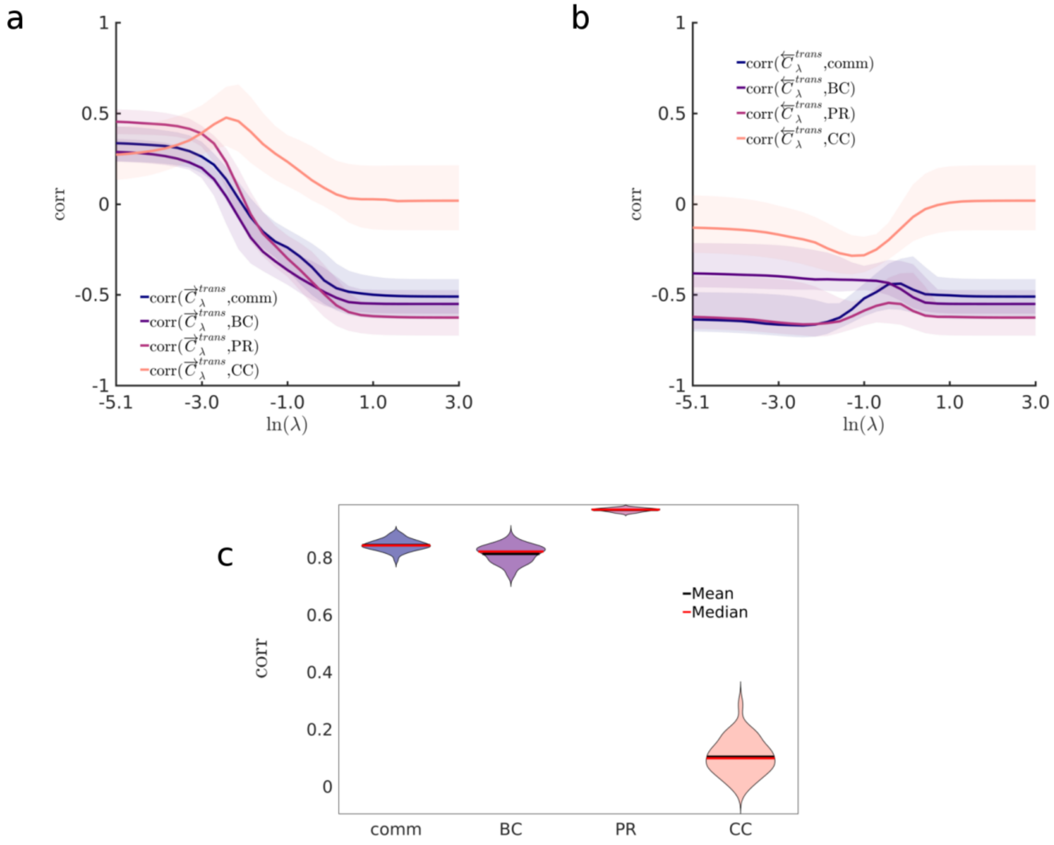

Comparison between the nodal C_λ^{trans} measures ($\vec{C}_\lambda^{trans}$ and $\overleftarrow{C}_\lambda^{trans}$) computed on the LAU dataset, and communicability (comm), betweenness centrality (BC), page rank centrality (PR) and clustering coefficient (CC).
