# Supplementary material for: A spectrum of routing strategies for brain networks
Source: PLoS Comput Biol. 2019 Mar 8;15(3):e1006833. doi: 10.1371/journal.pcbi.1006833 (PMC6426276; doi:10.1371/journal.pcbi.1006833)
Supplement: S2 Text — (PDF) [file pcbi.1006833.s011.pdf]

S11 Supporting Information. Testing the effect of binarizing the structural connectivity networks.

In order to test the significance of including the weights of the connections of the data in our analyses, we have replicated our results with the HCP cohort by binarizing each subject's structural network. Our findings show that the statistics computed at a global level (i.e. averaging across all nodes) are qualitatively similar to the results obtained when the weights of the connections are included. However, we find that there are significant differences in the statistics computed at the nodal and pair-wise level, with pair-wise differences being greater for lower values of λ. The following figure shows average global and nodal C_{λ}^{trans} and C_{λ}^{info} measures computed across all subjects of the HCP dataset after binarizing the networks. We also show scatter plots of the pair-wise C_{λ}^{trans} measures of the binarized and weighted networks, demonstrating that the results change at the local level with the inclusion or exclusion of the connection weights.

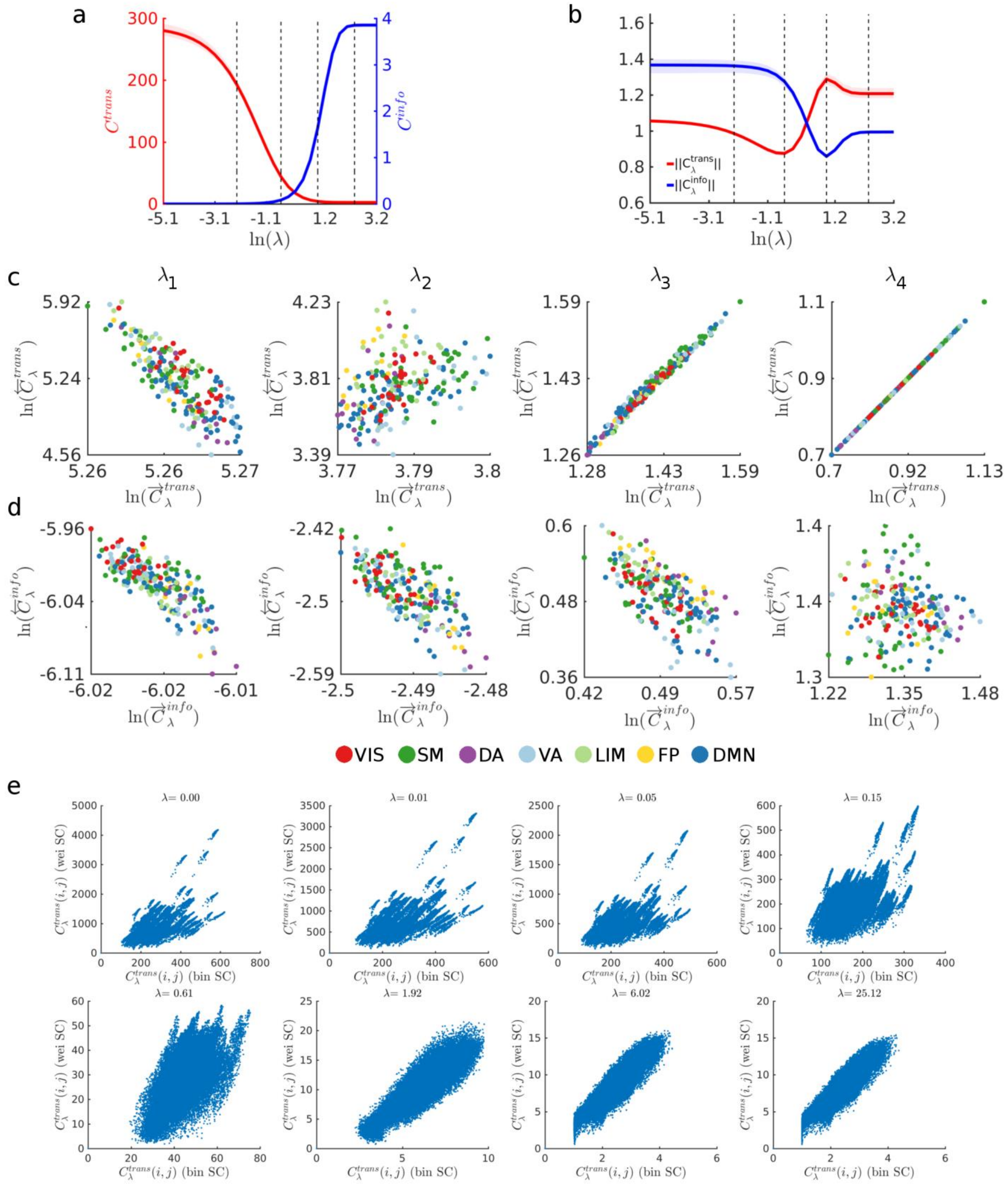

Averages of C_λ^{trans} (red) and C_λ^{info}(blue) across all node pairs, as a function of λ. Solid red and blue lines correspond to the median across all subjects, whereas the shaded red and blue regions denote the 95th percentile. (b) Averages of $\|C_\lambda^{trans}\|$ (red) and $\|C_\lambda^{info}\|$ (blue) across all node pairs. These curves are computed by normalizing C_λ^{trans} and C_λ^{info} with respect to the same cost measures computed on

ensembles of 500 randomized networks (per subject). Sections of the curves $\|C_\lambda^{trans}\|$ and $\|C_\lambda^{info}\|$ that are smaller than 1, respectively, indicate the regions in the spectrum where the communication cost of unweighted empirical networks (i.e. networks that are derived from empirical data) is smaller than the cost computed on the unweighted randomized ensembles. The dashed vertical lines are placed at the minimum and maximum of $\|C_\lambda^{trans}\|$ (λ_2 and λ_3, respectively), and at two points near the extremes of the spectrum ((λ_1 and λ_4). (c) Nodal average transmission costs for four increasingly biased routing strategies computed on the unweighted structural networks. Scatter plots show the transmission cost associated to each node when it acts as source ($\vec{C}_\lambda^{trans}$) and target ($\overleftarrow{C}_\lambda^{trans}$) during communication processes taking place under routing strategies generated with the values λ_1, λ_2, λ_3 and λ_4. (d) Scatter plots show the informational cost associated to each node when it acts as source ($\vec{C}_\lambda^{info}$) and target ($\overleftarrow{C}_\lambda^{info}$) during communication processes taking place under routing strategies generated with the values λ_1, λ_2, λ_3 and λ_4. Markers representing each node, are colored according to the node's membership in the 7 intrinsic connectivity networks (ICN) defined by Yeo et al. (2011) [71]: Visual (VIS), Somatomotor (SM), Dorsal Attention (DA), Ventral Attention (VA), Limbic (LIM), Frontal Parietal (FP), and Default Mode Network (DMN). The size of the markers is proportional to node's degree. (e) Scatter plots of the pair-wise C_λ^{trans}, averaged across all subjects, and computed on the unweighted (x-axis) and weighted (y-axis) structural networks.
